# Estimation of glomerular filtration rate in paediatric cancer patients using ^51^CR-EDTA population pharmacokinetics

**DOI:** 10.1038/sj.bjc.6601484

**Published:** 2004-01-06

**Authors:** M Cole, L Price, A Parry, M J Keir, A D J Pearson, A V Boddy, G J Veal

**Affiliations:** 1 Northern Institute for Cancer Research, Medical School, University of Newcastle, Newcastle upon Tyne NE2 4HH, UK; 2 School of Clinical Medical Sciences (Child Health), University of Newcastle, Newcastle upon Tyne NE2 4HH, UK; 3 Department of Medical Physics, University of Newcastle, Newcastle upon Tyne NE2 4HH, UK; 4 School of Mathematics and Statistics, University of Newcastle, Newcastle upon Tyne NEI 7RU, UK

**Keywords:** renal function, population pharmacokinetics, paediatric, cancer

## Abstract

Estimation of glomerular filtration rate (GFR) using the clearance of chromium 51 EDTA (^51^Cr-EDTA) (or other radiolabelled isotopes) is reliable, but invasive and not always practicable. Mathematical models have been devised for estimating GFR using readily obtainable patient characteristics. Unfortunately, these models were developed using various patient populations and may not provide the optimal prediction of GFR in children with cancer. The current study uses population pharmacokinetics to determine the relationship between ^51^Cr-EDTA clearance, and patient covariates in 50 paediatric cancer patients. These models were validated using a separate group of 43 children and were compared with previously published models of renal function. Body size was the major determinant of ^51^Cr-EDTA clearance and inclusion of weight or surface area reduced the residual variability between individuals (coefficient of variation) from 61 to 32%. Serum creatinine was the only other parameter that significantly improved the model. Mean percentage error values of –5.0 and –1.1% were observed for models including weight alone or weight and creatinine, respectively, with precision estimates of 21.7 and 20.0%. These simple additive models provide a more rationale approach than the use of complex formulae, involving additional parameters, to predict renal function.

Assessment of the glomerular filtration rate (GFR) is widely accepted as the best index of renal function in patients. Many chemotherapeutic drugs are excreted to a large extent via the kidneys, thus a reliable and accurate measurement of this parameter is particularly important in oncology practice. The relationship between kidney function and the extent of drug exposure is best exemplified by carboplatin, where the dose of drug administered is determined by renal function in both adult and paediatric patients (Calvert *et al*, 1989; Newell *et al*, 1993; Thomas *et al*, 2000). In addition, a measurement of renal function may be important in monitoring the nephrotoxic effects of drugs such as cisplatin and ifosfamide (Skinner *et al*, 1994).

An accurate determination of GFR can be obtained by measuring the clearance of chromium 51 EDTA (^51^Cr-EDTA) or similar isotope-based methods (Chantler *et al*, 1969; Rodman *et al*, 1993). This approach would be recommended when an accurate prediction of GFR is required, particularly in patients with reduced renal function. However, EDTA is not licenced for use in countries such as the USA. Alternatively, GFR can be estimated from serum creatinine concentration or calculated creatinine clearance (Perrone *et al*, 1992). These latter methods offer a less precise estimation of renal function, but can generally be performed with minimum patient inconvenience and at a lower cost than the isotopic methods. In paediatrics, problems may arise as these methods have commonly been validated in adults and it is difficult to obtain an accurate collection of urine over a 24-h period. A noninvasive, simple and reliable mathematical model for predicting GFR in a paediatric patient population would be advantageous, particularly if the withdrawal of multiple blood samples could be avoided.

Several mathematical equations and nomograms have been developed to predict renal function (Jelliffe, 1973; Cockcroft and Gault, 1976; Schwartz *et al*, 1976). The most commonly used of these formulae to predict creatinine clearance, and hence GFR, in adult patients have been those published by Cockcroft and Gault and that of Jelliffe, using age, sex, serum creatinine and either body weight (Cockcroft and Gault) or surface area (Jelliffe) as a measure of body size. In paediatric patients, perhaps the most widely used is that described by Schwartz, which is based on the ratio of height to serum creatinine concentration and includes an adjustment for patient age (Schwartz *et al*, 1976).

Whereas in adult patient populations these formulae approximate GFR to an acceptable level (Luke *et al*, 1990), similar studies in paediatric patient populations have highlighted inaccuracies. In a study involving patients aged between 2 and 18 years, 95% of GFR estimates obtained from the Schwartz formula would be expected to lie within 50% of the GFR determined as the clearance of ^51^Cr-EDTA (Skinner *et al*, 1994). In clinical practice, a higher level of accuracy is often required and the use of these models has been associated with inaccurate dosing of carboplatin (Calvert and Egorin, 2002).

The statistical methodology routinely used to model ^51^Cr-EDTA pharmacokinetics does not take into account the sampling variability in the estimates of ^51^Cr-EDTA clearance. For this reason, approaches based on nonlinear mixed effects models, often referred to as population models, have recently been published and independently assessed (Martin *et al*, 1998; Wright *et al*, 2001; Poole *et al*, 2002; Léger *et al*, 2002). These methods involve the use of patient specific ^51^Cr-EDTA plasma data together with supplementary covariate information. The current study applies nonlinear mixed effects modelling to the pharmacokinetics of ^51^Cr-EDTA, with a view to predicting GFR in paediatric cancer patients.

## MATERIALS AND METHODS

### Patients

Data were collected retrospectively on a total of 93 patients (50 male, 43 female) diagnosed between 1990 and 2000 and treated for cancer at the Royal Victoria Infirmary in Newcastle upon Tyne, UK. All of the patients had undergone assessment of renal function via ^51^Cr-EDTA clearance; a blood sample for biochemical analysis had also been taken at the same time. Biochemical analysis included the determination of levels of sodium, potassium, urea, albumin, total protein and bilirubin. The Boyd formula (Boyd, 1935) as recommended by Sharkey *et al* (2001) was used to estimate body surface area in this study.

There were a wide variety of malignancies including 35 bone, 26 soft-tissue sarcoma, 13 neuroblastoma and 10 germ cell tumours (Table 1

**Table 1:** Summary of patient characteristics

**Variable**	**Modelling data set**	**Validation data set**
Number of patients	50	43
Number <1 year	2	0
Number <10 kg	3	3
Sex (male/female)	26/24	24/19
*Diagnosis*		
Bone	22	13
Carcinoma	1	1
Germ cell	3	7
Neuroblastoma	6	7
Soft-tissue sarcoma	14	12
Other	4	3

). None of the patients had undergone a nephrectomy. Ages ranged from 10 months to 19.8 years.

### ^51^Cr-EDTA clearance data

Following an intravenous bolus administration of ^51^Cr-EDTA, three plasma samples were taken at approximately 1, 2 and 4 h. The first sample was taken at a median time of 67 min (range, 55–135 min), the second at 129 min (109–247 min) and the third at 247 min (187–395 min).

^51^Cr-EDTA clearance was assumed to be an accurate measure of GFR and is subsequently referred to as such. Glomerular filtration rate was estimated by fitting a linear least-squares regression to the logarithmically transformed counts per minute per ml (c.p.m. ml^−1^) *vs* time data. Assuming a one-compartment model for ^51^Cr-EDTA pharmacokinetics, the elimination rate constant (*k*) is estimated by the negative of the slope of the regression line, while the volume of distribution (*V*_d_) is estimated by the ratio of the injected c.p.m. to the estimated c.p.m. ml^−1^ at time zero. Glomerular filtration rate is calculated as the product of *k* and *V*_d_.

### Population models for ^51^Cr-EDTA

Of the 93 patients, 50 were randomly assigned to a modelling data set used to derive the population model for ^51^Cr-EDTA; the remaining 43 were assigned to a validation data set used to assess the ability of the models to predict GFR. Data from patients assigned to the validation set were not used to derive the population model.

The development of a population model for ^51^Cr-EDTA was undertaken using the first-order conditional estimation method implemented as a part of the NONMEM Version V level 1.1 software (Boeckmann *et al*, 1997). The following general modelling strategy was employed; however, there was inevitably an iterative element to the process: (i) appropriate structural and error models were determined; (ii) models to account for variation in clearance due to ‘body size’ were examined; and (iii) a forward selection strategy was used to account for variation in clearance not accounted for by ‘body size’; covariates considered were sex, sodium, potassium, urea, albumin, total protein and bilirubin. Choices concerning model structure and covariate selection were based on the reduction in NONMEM objective function value and interindividual variability, together with the examination of residual plots. Finally, the functional forms of other clearance models based on previously published studies were investigated.

### Model validation

Glomerular filtration rate was estimated for each individual in the validation data set using each of the candidate ^51^Cr-EDTA population clearance models and each of the previously published GFR estimation methods. The percentage error and precision of the various methods, relative to GFR as determined by the standard ^51^Cr-EDTA clearance method, were assessed by the mean percentage error (MPE) and mean absolute percentage error, respectively. The limits of agreement were calculated for the percentage error (Bland and Altman, 1986).

## RESULTS

### Population models for ^51^Cr-EDTA

There were no apparent differences between the ‘modelling’ and ‘validation’ groups in terms of age, weight and height profiles, or in the distribution of the biochemical measurements (Tables 1 and 2

**Table 2:** Comparison of modelling and validation sets with regard to all covariates

	**Modelling data set**		**Validation data set**		**All patients**	
**Variable**	**Median**	**Range**	**Median**	**Range**	**Median**	**Range**
Age (years)	10.5	(0.8–19.8)	8.7	(1.0–19.3)	10.3	(0.8–19.3)
Weight (kg)	33.2	(8.4–93.7)	25.0	(8.0–80.0)	31.1	(8.0–93.7)
Height (cm)	144	(72–194)	130	(74–191)	142	(72–194)
Body surface area (m^2^)	1.12	(0.44–2.25)	0.92	(0.42–2.02)	1.07	(0.42–2.25)
Serum creatinine (*μ*mol l^−1^)	59.5	(32–99)	57	(24–89)	58	(24–99)
Sodium (mmol l^−1^)	139	(130–145)	139	(126–143)	139	(126–145)
Potassium (mmol l^−1^)	4.2	(3.3–5.0)	4.2	(3.6–5.5)	4.2	(3.3–5.5)
Urea (mmol l^−1^)	3.8	(1.5–6.8)	4.0	(1.5–7.0)	3.9	(1.5–7.0)
Albumin (g l^−1^)	41	(27–48)	41	(25–50)	41	(25–50)
Protein (g l^−1^)	68	(57–80)	65	(50–78)	68	(50–80)
Bilirubin (*μ*mol l^−1^)	8	(2–20)	8	(3–51)	8	(2–51)
^51^Cr-EDTA clearance (ml min^−1^)	74	(14–198)	80	(12–178)	76	(12–198)

). Median GFR was 76 ml min^−1^ with a range of 12–198 ml min^−1^ (Table 2), and the frequency distribution of GFR in all the patients studied is given in Figure 1

**Figure 1:**
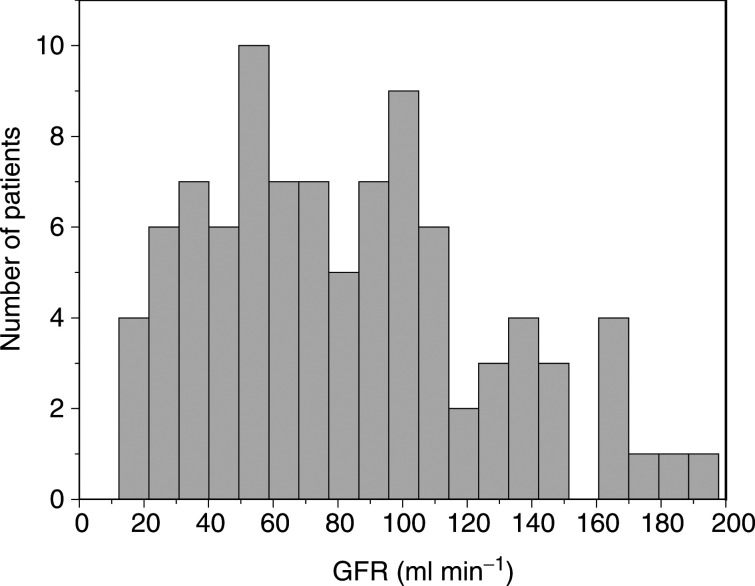
Distribution of GFR in all patients studied.

.

^51^Cr-EDTA pharmacokinetics were adequately described by a one-compartment model, intrasubject variation by an additive error model and between-subject variation in both clearance and *V*_d_ by an exponential error model.

Clearance of ^51^Cr-EDTA was strongly related to body size. The coefficient of variation (CV) in the between-subject error for clearance was reduced from 61 to 32% when either BSA or weight was included in the model. Various transformations of BSA and weight were considered, for example logarithmic, but none of these improved the fit of the model beyond a simple additive term. Weight, rather than BSA, was retained in the model (Table 3

**Table 3:** Formulae to predict GFR (ml min^−1^)

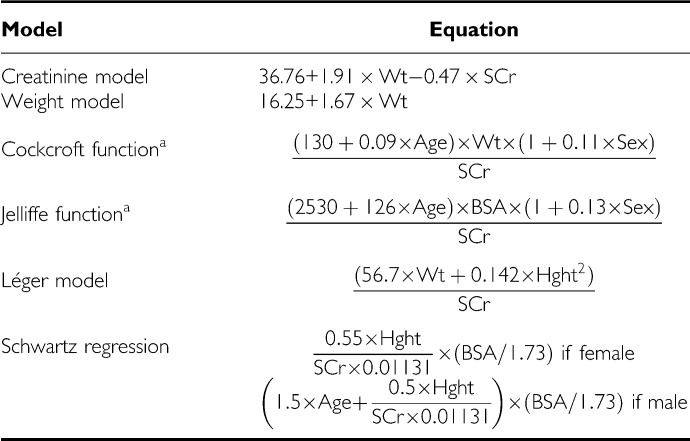

Coefficients derived from modelling data set, except for Schwarz and Léger equations where the original coefficients are used. Wt: weight (kg); Age: age (years); Sex: 1 if male, 0 if female; SCr: serum creatinine (*μ*mol l^−1^); BSA: body surface area (m^2^); Hght: height (cm).

^a^Coefficients re-estimated from current data set using nonlinear mixed effects modelling.

) as estimates of BSA are often based on weight and height, with height being difficult to estimate in small children (Sharkey *et al*, 2001).

None of the remaining potential covariates significantly reduced the CV in the between-subject error for clearance, with the exception of serum creatinine. Various transformations for serum creatinine were considered, but a simple additive term again proved to be sufficient (Table 3), resulting in a further reduction of the CV to 28%. At this stage none of the other variables, including age and sex, improved the fit of the model. Although serum bilirubin had the next biggest impact on improving the fit, the addition of this variable was far from statistically significant.

### Comparison of models

The various models for ^51^Cr-EDTA clearance used to assess predictive performance are shown in Table 3. The ‘weight’ and ‘creatinine’ models are the population models obtained from this study. The equations labelled ‘Cockcroft function’ and ‘Jelliffe function’ were obtained by using the functional form of previously published models that were derived in adults. As applied here, the coefficients were re-estimated using the current modelling data set. The coefficients in the Schwartz and Léger models are those that have been published previously, as these were derived from a paediatric population.

The ‘weight’ model predicts an increase in GFR of 1.67 ml min^−1^ for a 1 kg increase in body weight. For a ‘typical’ 10 kg child this relates to a 5% increase in GFR, and a 1% increase for a ‘typical’ 70 kg child. Similarly, given a 5 *μ*mol l^−1^ increase in serum creatinine, the ‘creatinine’ model predicts a 7% decrease in GFR for a 10 kg child with a serum creatinine of 45 *μ*mol l^−1^ (roughly the median serum creatinine level for a child of this weight). For a larger 70 kg child with a serum creatinine of 75 *μ*mol l^−1^, the ‘creatinine’ model predicts a 2% decrease in GFR for the same 5 *μ*mol l^−1^ increase in serum creatinine.

A comparison of the percentage error of the various formulae using the validation set (together with the limits of agreement) is presented in Table 4

**Table 4:** Comparison of formulae percentage error and limits of agreement

**Percentage error**	**Mean (95% confidence interval)**	**95% Limits of agreement**
Creatinine model	−1.1 (−8.9, 6.8)	−50, 48
Weight model	−5.0 (−13.3, 3.2)	−56, 46
Cockcroft function	3.4 (−4.3, 11.0)	−44, 51
Jelliffe function	1.2 (−6.1, 8.4)	−44, 46
Léger	0.0 (−6.8, 6.9)	−43, 43
Schwartz regression	−5.8 (−13.3, 1.7)	−52, 41

**Figure 2:**
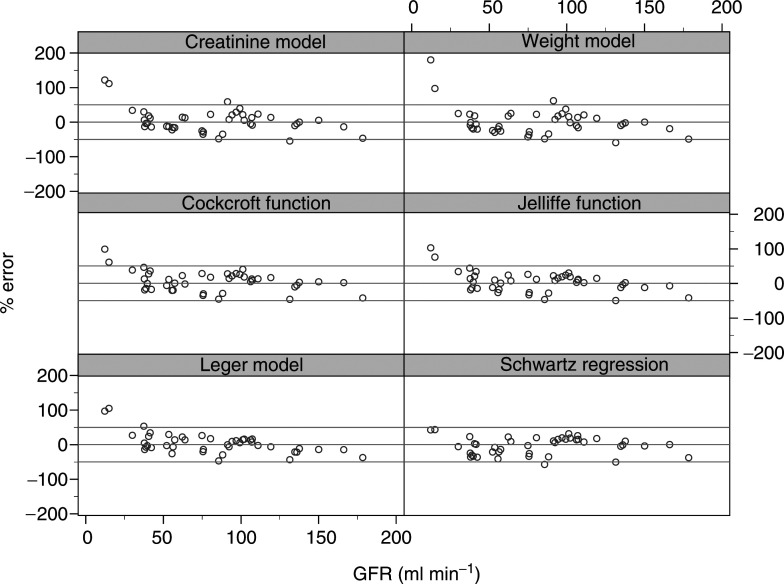
Comparison of formulae percentage error *vs* GFR as estimated by the clearance of ^51^Cr-EDTA.

Percentage error is defined as 100[(estimated GFR–actual GFR)/(actual GFR)]. The actual GFR is obtained from the clearance of ^51^CR-EDTA. In all, 95% of values would be expected to lie within the limits of agreement.

; 95% of values would be expected to lie within these limits. Figure 2

shows the percentage error plotted against GFR for each equation. Two patients who had GFRs less than 25 ml min^−1^ were excluded from the statistical analysis of the validation set, although their data are shown in Figure 2.

The Léger, Jelliffe and the creatinine models have the smallest percentage error, although the 95% confidence intervals are relatively wide. The Schwartz and weight models tend to underestimate, while the Cockcroft model tends to overestimate; however, none of the models showed a statistically significant percentage error. Also, none of the models have a statistically significantly smaller percentage error than any other model (data not shown). The 95% limits of agreement are wide in each case, the typical range being –50%, 50%. This implies that 5% of estimated GFRs would be expected to differ from the true value by more than 50%.

**Figure 3:**
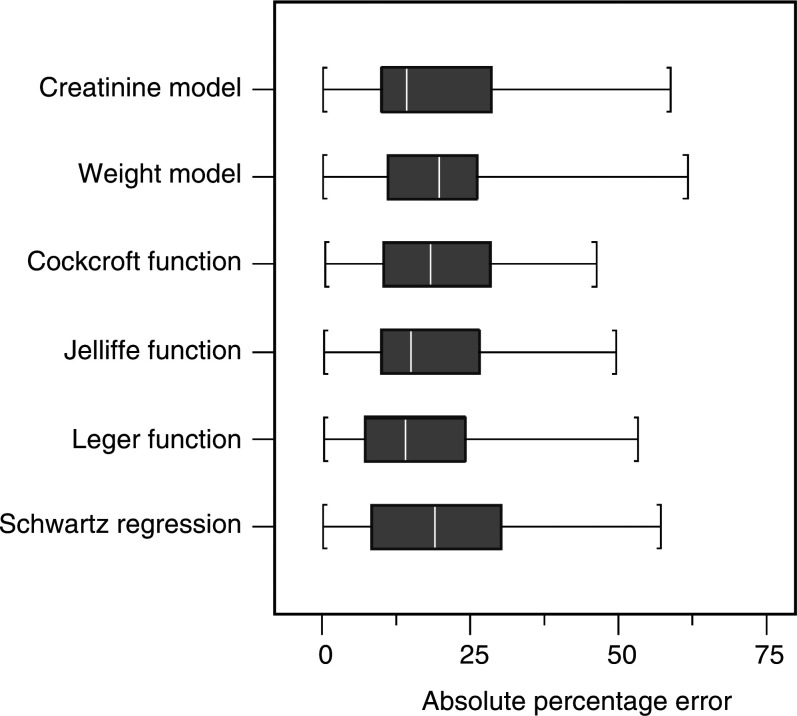
Comparison of formulae precision, defined as the absolute percentage error. Boxplot showing absolute percentage error, excluding individuals with GFR <25 ml min^−1^.

The precision of the various equations is shown graphically in Figure 3

. Again, there is no significant difference between the various models, with median precision values of approximately 20%.

## DISCUSSION

An accurate estimation of renal function in children is important in optimising the dose of many drugs used in paediatric oncology, and allows clinical monitoring of the nephrotoxic effects of cytotoxic agents such as cisplatin. The current study investigates the relationship between ^51^Cr-EDTA population pharmacokinetics and patient covariates commonly obtained from children with cancer. Population models for ^51^Cr-EDTA clearance were derived from 50 paediatric patients randomly assigned to a modelling data set and were validated using a separate group of 43 children. The abilities of these models to predict GFR were compared with the functional form of previously published creatinine clearance models. This type of population pharmacokinetic approach for predicting GFR has previously been used successfully in adults (Martin *et al*, 1998; Wright *et al*, 2001) and in children with renal disease (Léger *et al*, 2002).

^51^Cr-EDTA clearance was strongly correlated to body size, with a simple additive model (16.25+1.67 × Wt) proving to be as good a fit as that obtained using more complex transformations of BSA and weight. Of the remaining parameters, only serum creatinine was seen to improve the model, as determined by a significant reduction of the CV in the between-subject error for clearance. Again, a simple additive term proved to be as efficient in predicting GFR as any more complex transformations, giving rise to a refined model (GFR=36.76+1.91 × Wt−0.47 × SCr). Owing to the significant variation in body weight in the patient population studied (range: 8–93.7 kg), and the dominant effect of this covariate, other variables, such as age and sex, failed to improve the fit of the model. Bilirubin was shown to be the next biggest factor, but did not significantly improve the fit of the model. Serum creatinine was highly correlated with weight (data not shown), such that the predictive power of this covariate was compromised in this patient group.

The application of the population models developed in this study to the validation patient group resulted in an MPE of −5.0 and −1.1% for the ‘weight’ and ‘creatinine’ models, respectively. Precision estimates of 21.7 and 20.0% were obtained for these two models, indicating that the use of creatinine in addition to weight results in a marginally more robust prediction of ^51^Cr-EDTA clearance. Adopting the functional forms of established and commonly used equations, but with coefficients re-estimated using a paediatric data set, there was little to choose between the various models in terms of estimated MPE and precision. Slight variations in the percentage error (mean values ranging from ±0.0 to 5.8%) and precision (mean values ranging from 17.5 to 21.7%) are overshadowed by the wide 95% limits of agreement observed for all the models investigated, with typical ranges of –50 to 50%. These results imply that 5% of estimated GFRs would be expected to be more than 50% discrepant from the true value, are not dissimilar to those noted previously using the Schwartz equation and highlight the shortcomings of all the models studied in this patient population.

Detailed analysis of the current data failed to identify subpopulations of patients, such as those with unusually high or low GFR values, in whom a particular model may be more appropriate. Two patients with very low GFR values (12 and 15 ml min^−1^) were excluded from the analysis of the validation set. These patients were both under 2 years of age weighing 11 and 8 kg, respectively. Their serum creatinine levels were 64 and 43 *μ*mol l^−1^. All of the equations predicted their GFR poorly, substantially overestimating in each case. For example, the Cockcroft model predicts 24 ml min^−1^ for both patients, an overestimate of 99 and 61%. For patients with estimated GFR values less than 40 ml min^−1^, it is advisable to obtain a direct determination of renal function using the ^51^Cr-EDTA, or similar, method.

The data presented here, and the models derived from them, are specific to paediatric cancer patients. Nevertheless, there are limitations to the use of any of these equations in clinical practice. Firstly, prospective prediction of GFR using these equations is unlikely to be as good as in the study in which they were developed. The models should be used with caution in subgroups of patients not included in the original validation. In addition, using prediction equations based on serum creatinine concentration can be problematic if creatinine concentrations are not at steady state (Payne, 1986; Perrone *et al*, 1992). Although the use of serum cystatin C as a parameter for predicting renal function has been recently suggested (Stabuc *et al*, 2000; Dharnidharka *et al*, 2002), its use in paediatric patients appears to offer few advantages over that of serum creatinine (Krieser *et al*, 2002; Willems *et al*, 2003). While cystatin C measurements were not obtained as a part of the current study, it seems unlikely that its use would have made a significant difference to the results obtained.

If a noninvasive estimate of GFR is required in children with cancer, the formula involving weight and serum creatinine as the only two variables performs as well as any of the equations that are commonly used in predicting renal function. Using a linear form of equation is more rational than the use of more complex formulae. For situations in which an accurate measurement of renal function is essential, the determination of GFR as clearance of ^51^Cr-EDTA is strongly recommended. Such situations include using an estimation of GFR to guide the dosing of high-dose carboplatin chemotherapy, which can result in severe and potentially life-threatening side effects, and dealing with children with a reduced renal function.
